# Principal Components Analysis of Population Admixture

**DOI:** 10.1371/journal.pone.0040115

**Published:** 2012-07-09

**Authors:** Jianzhong Ma, Christopher I. Amos

**Affiliations:** Department of Genetics, The University of Texas MD Anderson Cancer Center, Houston, Texas, United States of America; Medical College of Wisconsin, United States of America

## Abstract

With the availability of high-density genotype information, principal components analysis (PCA) is now routinely used to detect and quantify the genetic structure of populations in both population genetics and genetic epidemiology. An important issue is how to make appropriate and correct inferences about population relationships from the results of PCA, especially when admixed individuals are included in the analysis. We extend our recently developed theoretical formulation of PCA to allow for admixed populations. Because the sampled individuals are treated as features, our generalized formulation of PCA directly relates the pattern of the scatter plot of the top eigenvectors to the admixture proportions and parameters reflecting the population relationships, and thus can provide valuable guidance on how to properly interpret the results of PCA in practice. Using our formulation, we theoretically justify the diagnostic of two-way admixture. More importantly, our theoretical investigations based on the proposed formulation yield a diagnostic of multi-way admixture. For instance, we found that admixed individuals with three parental populations are distributed inside the triangle formed by their parental populations and divide the triangle into three smaller triangles whose areas have the same proportions in the big triangle as the corresponding admixture proportions. We tested and illustrated these findings using simulated data and data from HapMap III and the Human Genome Diversity Project.

## Introduction

The recent development of high-density single-nucleotide polymorphism (SNP) genotyping assays has made it possible to characterize patterns of genetic variation within and among human populations, providing unprecedented opportunities to understand the evolutionary history and migration patterns of humans. For genetic epidemiologists, it is critical to quantify population structure and admixture to enable careful study design and correction for population stratification. Principal components analysis (PCA) is widely used to quantify patterns of population structure [1]–[8]. The Eigenstrat method, as implemented in the program SmartPCA [1], [2], is now routinely used to detect and correct for population stratification in genome-wide association studies (GWAS). In conventional PCA, in which the markers are treated as features, sampled individuals are projected into a subspace spanned by the top principal components (PCs). Because the top PCs reflect variations due to population structure in the sample, individuals from the same population are found to form a cluster in this subspace. Therefore, the pattern of the scatter plot of the top PCs (PC-plot) is used to infer population relationships or within-population structures.

Although this relationship between the pattern of a PC-plot and population structure can be understood intuitively, it remains a challenging task to make appropriate and correct inferences about population structure from PC-plots. Usually, the genetic similarities or dissimilarities of populations are inferred from the Euclidean distances between their clusters in the PC-plot. This metric, however, is not always reliable. For example, the PC-plot pattern is strongly influenced by the relative sample size of the populations. Admixture induces further complications in analyzing population structures from PCA results. For simulated data, it is found that admixed individuals are dispersed along the line segment connecting the clusters of the two parental populations in the two-dimensional space of the first two PCs. This kind of dispersion along a line has been used as a diagnostic of admixture [1]. The relative distances of an individual from the centroids of the clusters of the parental populations have been used to estimate the admixture proportions of the individual [9], [10]. However, this kind of dispersion may be difficult to distinguish from scattering due to sampling fluctuations or within-population variations. More importantly, it is not clear how three-way or multi-way admixture manifests itself in the PC-plot pattern, and how the admixture proportions can be inferred.

In a recent publication [6], we developed a theoretical formulation of PCA in which sampled individuals from different populations are treated as features and the markers as samples or realizations. Because each individual is represented by a fixed position of the eigenvectors of the variance-covariance matrix, the pattern of the plot of the top eigenvectors reflects the population structure, just as does the PC-plot in conventional PCA. Because individuals from different populations as features are predetermined, we were able to build a theoretical framework where parameters reflecting the population properties and relationships can be defined *a priori*. This is why we were able to directly relate the population parameters to the theoretical pattern of the eigenvector-plot, without having to deal with sampling fluctuations or within-population variations. Using this formulation, we were able to predict the pattern of eigenvector-plot from known population structures and thus appropriately infer population structures from the results of PCA [6]. We also used our formulation to quantitatively determine how the sample sizes influence the pattern of the eigenvector-plot. Specifically, we showed that there exists an asymptotically stable pattern of the eigenvector-plot when the overall sample size becomes large.

In this paper, we extend our theoretical formulation to allow for admixture. Our generalized formulation enables us to derive a set of reduced eigenequations that depends on the admixture proportions of admixed populations as well as the variance-covariance parameters of the parental populations and other populations. As in [6], an asymptotic form of the eigenequation can be derived from our formulation as the overall sample size becomes large, and it can be used to predict the influence on the pattern of the eigenvector-plot of the population parameters, admixture proportions, and the relative sample sizes of the populations. Specifically, using the generalized formulation, we first theoretically justify the diagnostic of admixture, namely a dispersion along the line connecting the two parental populations. We find that this diagnostic can be generalized to the case of three parental populations: admixed individuals are distributed inside the triangle formed by their parental populations and divide the triangle into three smaller triangles whose areas have the same proportions in the big triangle as the corresponding mixing proportions. We also extend this diagnostic to the case of an arbitrary number of parental populations. We demonstrate our theoretical findings using simulated data and data from HapMap III and the Human Genome Diversity Project (HGDP).

## Results

### General Framework

We first briefly outline the theoretical formulation of PCA developed in our previous publication [6]. The genotype data of individuals from 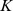 populations with sample sizes 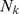 (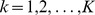) are modeled by a random vector 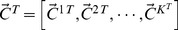, where 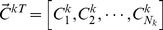 and 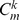 is the count of the variant allele for individual 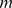 from population 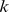 for a random marker. The randomness of each component comes from two sources: (i) the marker is randomly chosen, and hence the allele frequency is random, and (ii) the genotype, or the allele count (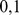, or 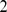), is determined randomly conditional on the allele frequency. In [6], we derived the following reduced eigenequation
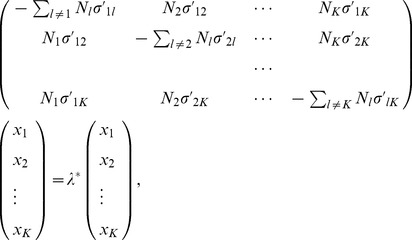
(1)where 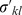 (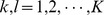) are the elements of the variance-covariance matrix, 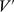, of the mean-adjusted allele counts:



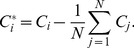
(2)Population structure is characterized by the 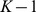 non-trivial eigenvectors obtained by solving Equation (1). Each of the coordinates of these 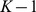 eigenvectors, 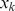 (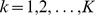), represents a population (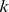) in the eigenvector-plot. In real data analysis, 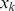 will be the centroid of the cluster formed by all individuals from population 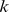. The pattern of the eigenvector-plot is determined by the variance-covariance parameters defined by 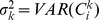 (the variance), 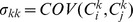 (the within-population covariance), and 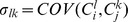 (the between-population covariance). The relationships between the 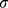
s and the 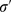
s are given in [6] (Equations (19–23)). These parameters, reflecting the population relationship, are related to the variance-covariance parameters
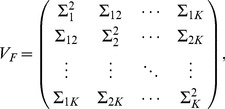
(3)of the random vector 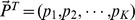 of the 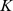 allele frequencies, by



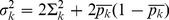
(4)

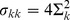
(5)

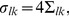
(6)where 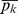 is the mean of 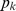, for 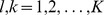 and 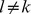.

In [6], the asymptotic form of the reduced eigenequation, in the limit of 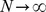, is given as follows:
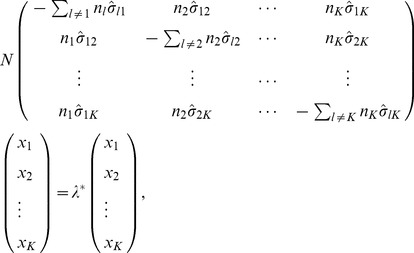
(7)where 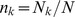 is the relative sample size of population 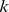, and
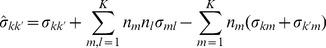
(8)for 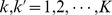 and 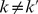.

### Modeling Admixture

To incorporate admixture into our theoretical formulation of PCA, we need to generalize Equations (4), (5), and (6) to the case where one or more populations are an admixture of some other populations. We begin with the case of two-way admixture. Suppose that there are five distinct populations: P1, P2, P3, P4, and P5. P3 and P4 are admixtures of P1 and P2 with proportions 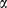: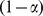 and 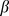: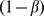, respectively. In Text S1, we show that the allele frequencies of the admixed populations, P3 and P4, can be expressed as
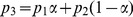
(9)

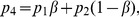
(10)and we derive the following expressions for the variance-covariance parameters of 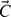 in terms of the expressions for the allele frequencies



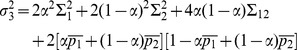


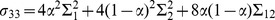


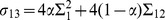


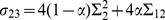
(11)

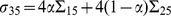


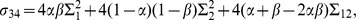
and similar expressions for population P4 with 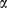 replaced by 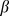. These listed parameters represent the variance in an admixed population (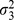), covariance within an admixed population (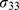), covariance between an admixed individual and the parental population (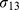 and 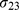), covariance between an admixed individual and an individual unrelated with admixture (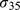), and covariance between an admixed individuals with different proportions (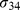). Other expressions for populations unrelated to admixture remain the same as in Equations (4), (5), and (6).

These expressions (11) can be generalized to the case with more than two parental populations. Suppose that there are six distinct populations, P1, P2, P3, P4, P5, and P6, among which P4 and P5 are admixed populations of P1, P2, and P3 with proportions 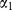: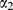: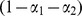 and 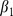: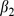: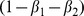, respectively. We have the following expressions (Text S1):
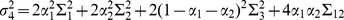


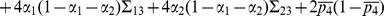


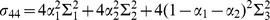


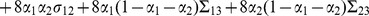


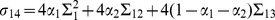


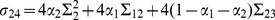
(12)

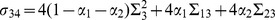


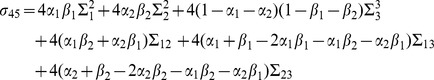


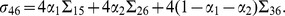



For the other admixed population, P5, we have similar expressions, except that the 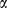
s are replaced by the corresponding 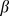
s. Details of the derivation of these expression are given in Text S1.

### Two-way Admixture

As shown in Text S2, a closed-form of solution can be obtained in the case of 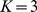 for both the reduced eigenequation (1) and its asymptotic form (7). Specifically, the two non-zero eigenvalues for Equation (7) are determined by
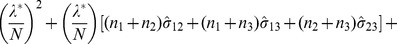


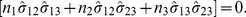
(13)


Now suppose that population P3 is an admixed population of the other two populations, P1 and P2, with admixture proportions 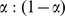. It can be shown, from Equations (11) and (8),
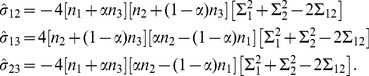
(14)


These expressions lead to an important sum-rule
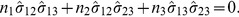
(15)


Using this sum-rule, we can see that the only non-zero eigenvalue is
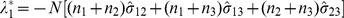
(16)and the corresponding eigenvector is



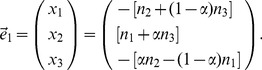
(17)We therefore have
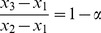
(18)

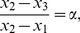
(19)which is the well-known relationship between the pattern of the eigenvector-plot and the admixture proportion. In Text S3, we show that the same conclusion, especially Equations (18) and (19), for calculating the admixture proportion from the coordinates of the eigenvector are still valid when the number of admixed individuals is not large. Therefore, this method of estimating admixture proportion can be applied to the individual level if there are sufficient samples from the parental populations.

**Figure 1:**
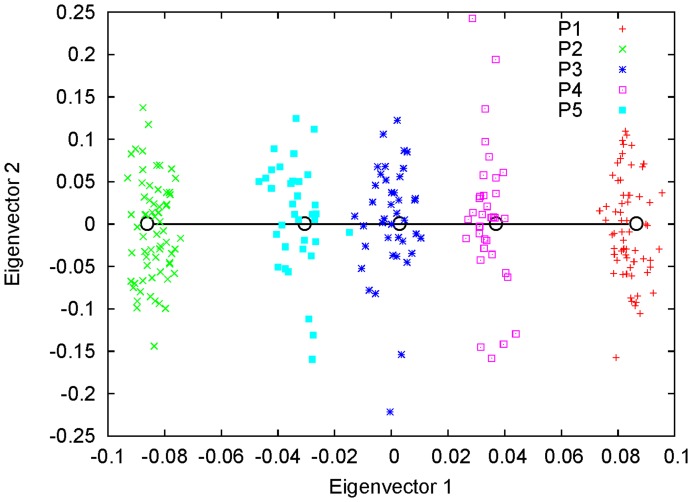
Simulation of genetically homogeneous admixed populations: two-way admixture. The first two eigenvectors are shown for a simulated data set with five populations. P1 and P2, the two parental populations of sample size 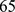 each, were both simulated using 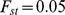. P3 is an admixture of P1 and P2 with proportion 0.5∶0.5. P4 is an admixture of P1 and P2 with proportion 0.3∶0.7. P5 is an admixture of P1 and P2 with proportion 0.7∶0.3. The sample size for the three admixed populations is 35. The ratios of the distances between the centroids of P3, P4, and P5 and those of P1 and P2 were found to be approximately equal to the corresponding admixture proportions.

The fact that there is only one non-zero eigenvalue reflecting the population structure implies that including an admixed population in PCA does not add to the number of axes of variation [1] if samples from the parental populations are already included in the analysis. The other non-trivial eigenvalue is 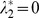 and the corresponding eigenvector is
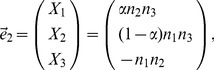
(20)where the coordinate of the admixed population 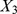 is outside of the interval of the coordinates of the two parental populations, 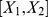. This means that in a two-dimensional eigenvector-plot with 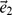 as one of the two axes, we cannot see a dispersion or gradient of the admixed individuals along the line segment connecting the centroids of the two parental populations. The second largest PC (corresponding to 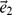) reflects within-population variation. The corresponding eigenvalue, 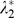, in the general case (when 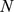 is not too large) is a small eigenvalue [6]. In Figure 1, we show an example of two-way admixture using simulation. Along the first eigenvector, the three admixed populations were distributed around some points between the two parental populations, and the ratios of the distances between the centroids of P3, P4, and P5 and those of P1 and P2, respectively, were found to be very close to the simulating values of the corresponding admixture proportions: 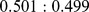, 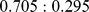 and 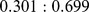. The centroids of all five populations were calculated by solving the reduced eigenequation (1) with variance-covariance parameters estimated using the method given in [6]. The distribution of individuals along the second eigenvector reflects within-population variations.

In order to have a two-dimensional pattern for which the admixed population is located on the slanted line segment connecting the centroid of the two parental populations, we need to introduce at least one additional population into PCA. This additional population is neither a parental population nor an admixed one. Adding the additional population to PCA introduces an additional dimension of variation, so that the first two PCs will be needed to address population structures. In this case, Equations (18) and (19) should be valid for the first two eigenvectors, although it does not seem to be easy to show it analytically. We demonstrated this numerically by solving Equation (1) in the case of 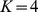 with P3 an admixed population of P1 and P2 (Figure S1).

**Figure 2:**
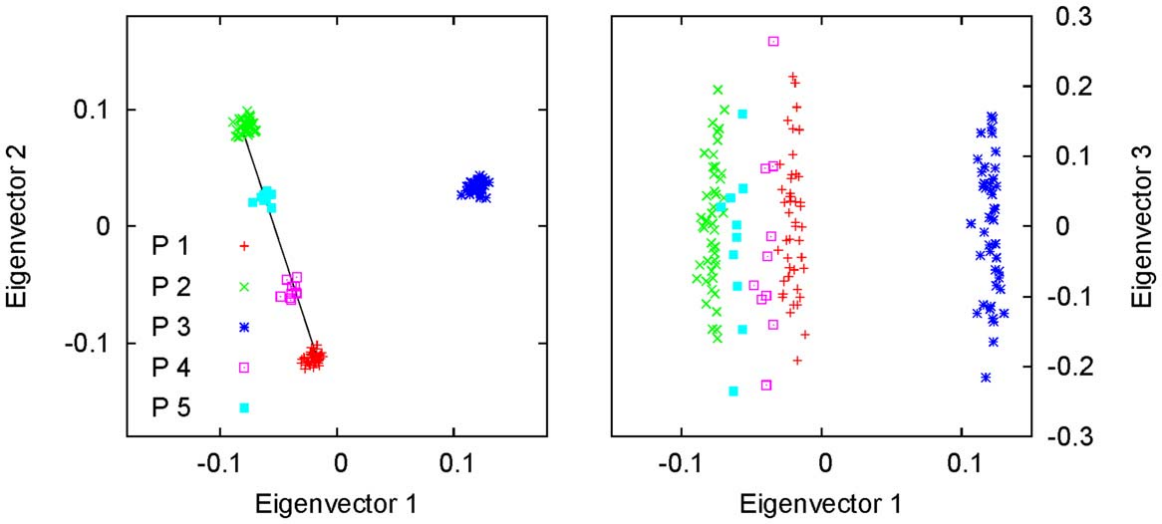
Simulation of genetically homogeneous admixed populations with an additional population: two-way admixture. The first two eigenvectors are shown for a simulated data set with five populations. P4 and P5, each with sample size 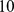, were simulated as admixed populations of P1 and P2 with admixture proportions 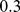: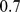 and 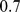: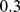, respectively. P3 was simulated as an additional population. P1, P2, and P3, each with sample size 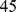, were simulated using 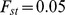. The clusters of P4 and P5 were found to lie on the line segment connecting the centroids of P1 and P2, and they divided the segment according to ratios that are approximately equal to the corresponding simulating values of the admixture proportions. The third eigenvector in the left panel addresses the within-population variations.

In Figure 2, we show an example of two-way admixture using simulation. P4 and P5 were two admixed populations, each with a sample size of 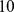, of populations P1 and P2 with admixture proportions 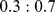 and 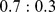, respectively. P3 was an additional population. P1, P2 and P3 were simulated with 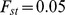, each with a sample size of 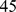. Here, we can see that the samples from the two admixed populations were located around the line segment connecting the centroids of the two parental populations in the space spanned by the first two eigenvectors: the distances of the centroids of P4 and P5 from the line segment were 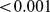, much smaller than the length of the line segment (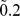). Again, the centroids of all five populations were calculated by solving the reduced eigenequation (1) with variance-covariance parameters estimated using the method given in [6]. The overall or average admixture proportions of P4 and P5 were estimated using Equations (18) and (19) as 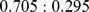 and 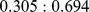, respectively. Along the third eigenvector, no population structure can be seen and only within-population variation was addressed. We applied the model-based clustering method, implemented in the program STRUCTURE, to P1, P2, P4, and P5 using the first 300 ancestry-informative markers (AIMs, see Methods), and found that the average admixture proportions were 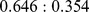 and 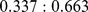 for P4 and P5, respectively.

**Figure 3:**
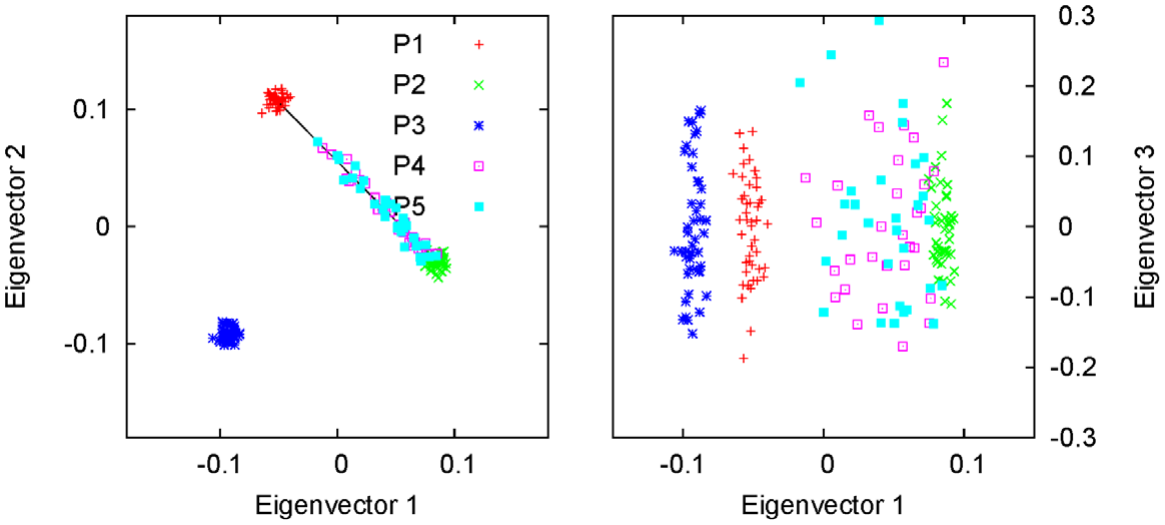
Simulation of genetically recently admixed populations with an additional population: two-way admixture. The first two eigenvectors are shown for a simulated data set with five populations. P4 and P5, with sample size 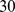, were simulated as admixed populations of P1 and P2 with admixture proportions drawn from a beta distribution with shape parameters 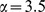 and 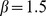. P3 was simulated as an additional population. P1, P2, and P3, each with sample size 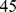, were simulated using 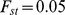. Samples from P4 and P5 were distributed along the line connecting the centroids of P1 and P2. Because there were only three independent populations (P1, P2, and P3), only two eigenvectors are needed to address the population variations. This is why along the third eigenvector, only the within-population variations were addressed.

In order to see a two-dimensional dispersion or gradient of admixed individuals distributed along the line segment connecting the centroids of the two parental populations, as often observed in real data analysis [1], the admixed population cannot be genetically homogeneous, as in the example shown in Figure 2. In other words, the admixed individuals need to have different admixture proportions. Figure 3 shows such an example. The simulation was the same as that in Figure 2, except that the individuals of P3 and P4 were simulated with admixture proportions drawn from a beta distribution with shape parameters 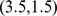. Here, we clearly see that the samples of P4 and P5 were restricted to the line segment connecting the centroids of of P1 and P2: the distances of the centroids of P4 and P5 from the line segment were both 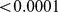. The individual admixture proportions of P4 and P5 were calculated using Equations (18) and (19) and were found to be very close to the corresponding simulating values (data not shown). Note that the admixed individuals of P4 and P5 were scattered along both directions on the right panel of Figure 3. However, the elongations of the two clusters along these two directions have different meanings: spreading along the first eigenvector implies a genetically recent admixture, whereas spreading along the third eigenvector simply reflects within-population variations.

For the example shown in Figure 3, the individual admixture proportions were also estimated using STRUCTURE for the two admixed populations, P4 and P5. As shown in Figure S2, our PCA approach outperformed STRUCTURE in the sense that the estimated admixture proportions obtained by PCA were much closer to the generating values than those obtained by STRUCTURE, although estimates from both methods were highly correlated (Spearman correlation coefficient 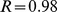).

**Figure 4:**
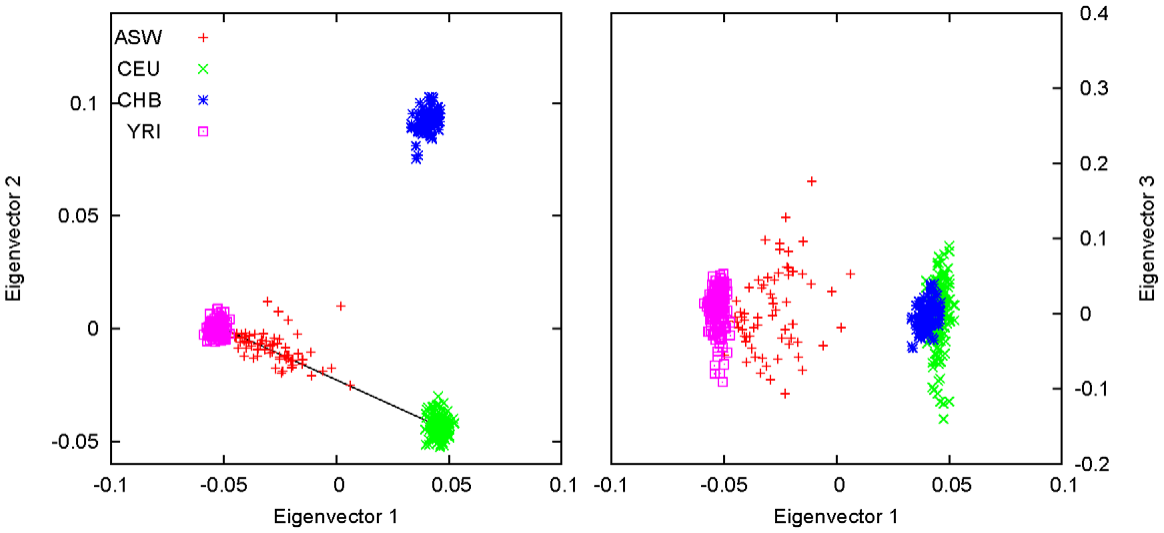
An example of two-way admixture from HapMap data. The first two eigenvectors are shown for the four HapMap populations ASW, CEU, CHB, and YRI. A dispersion, or gradient, was formed by the ASW samples as a recently admixed population. CEU and YRI served as the proxy parental populations of ASW. CHB was included in the analysis to introduce an additional dimension of variation, so that the dispersion can be seen in the two-dimensional space. The third eigenvector addresses the within-population variation.

Figure 4 shows the results for the four HapMap populations ASW, CEU, CHB and YRI using markers on chromosome 1, as an example of two-way admixture. Here, CEU and YRI were used to represent the European and African ancestries, respectively, of the ASW population. CHB was included in the analysis to provide an additional axis of variation. Most of the ASW samples were found to be restricted on the cline of CEU and YRI; the distance between the centroid of ASW and the line segment connecting the centroids of CEU and YRI is 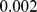, much smaller than the length of the line segment (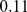). The results confirmed that the ASW population is a genetically recent admixture with an average of 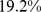 of European and 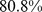 of African ancestry, as calculated using Equations (18) and (19) with P3 representing the centroid of ASW. The individual admixture proportions were calculated also using Equations (18) and (19) and are listed in Table S1. The results of STRUCTURE were also shown in Table S1. The average admixture proportions estimated by STRUCTURE were 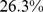 and 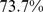, for European and African ancestry, respectively. The Spearman correlation coefficient between the results of PCA and those of STRUCTURE was 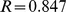.

### Three-way Admixture

**Figure 5:**
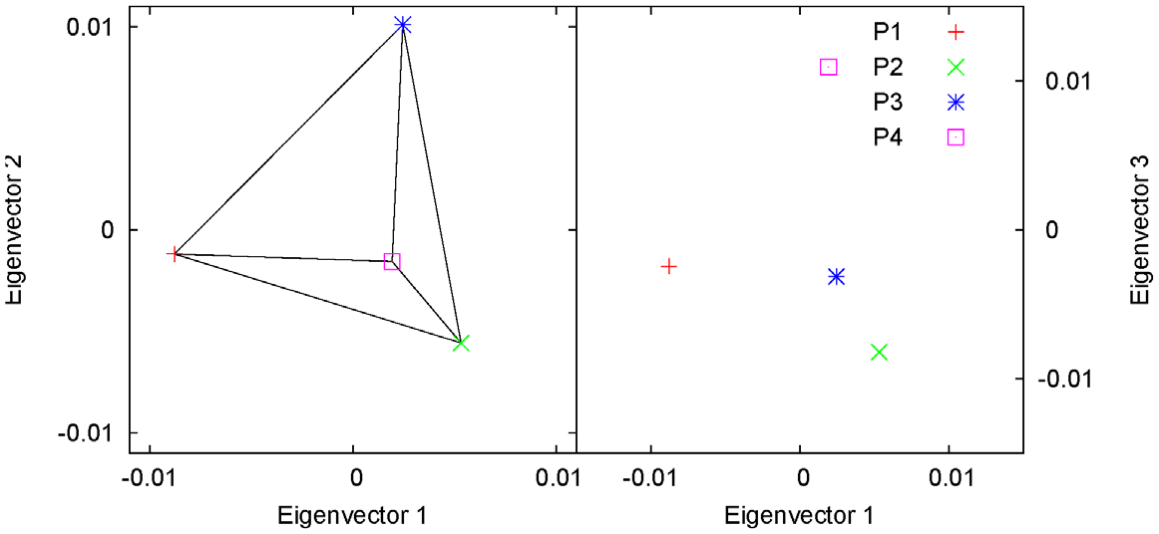
Theoretical prediction of PCA: three-way admixture. The eigenvectors for the four hypothetical populations defined in Table 1 were calculated from the reduced eigenequation (1). In the plane spanned by the first two eigenvectors (left panel), the representative point of the admixed population, P4, was located inside the triangle formed by the representative points of the three parental populations, P1, P2, and P3, and divided the triangle into three small triangles with areas according to the admixture proportions. On the right panel, P4 was outside the triangle because the third eigenvector, corresponding to a small eigenvalue, did not reflect population structure.

In the previous subsection, we have analytically proved the diagnostic of two-way admixture, that is, an admixed sample is located on the line segment connecting the two parental populations and divides the segment according to the admixture proportions. The proportion of ancestry from the parental populations can thus be estimated using the distances of the admixed sample from the parental populations in the eigenvector-plot. Natural questions arise: What is the diagnostic of three-way admixture? How can the three-way admixture proportions be estimated using the pattern of the eigenvector-plot? One of the main results of the present paper is as follows. Suppose that population P4 is an admixture of populations P1, P2, and P3 with admixture proportions 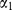: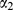: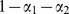. In the space spanned by the first two eigenvectors, the representative point of P4 is located inside the triangle formed by the representative points of P1, P2, and P3, and it divides the area of this triangle according to the admixture proportions:
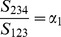
(21)

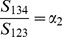
(22)

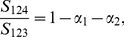
(23)where 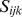 is the area of the triangle formed by P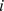, P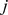, and P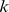. To prove Equations (21),(22) and (23) analytically, we would need to solve the reduced eigenequation (1) for 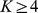 analytically. A closed-form of solution to this eigenequation, however, is difficult, if not impossible, to find. Nevertheless, we can numerically solve it. We confirmed Equations (21),(22) and (23) by numerically solving Equation :w (1) for various different combinations of variance-covariance parameters and admixture proportions when 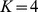. Figure 5 shows one example of a three-way admixture, for four hypothetical populations defined in Table 1. In Figure S3, we show an example of two three-way admixture, where it can also be seen that it is the two-dimensional metric (namely, the areas), instead of the one-dimensional metric (namely, the distances), that should be used to estimate the admixture proportions in the case of three-way admixture.

**Table 1:** Parameters for the hypothetical populations shown in Figure 5.

	P1	P2	P3	P4
Sample size	9000	8000	7000	10000
For allele count				
Variance ( 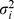 )	0.68	0.68	0.68	0.6224
Covariance ( 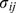 )				
P1	0.4	0.04	0.16	0.136
P2		0.4	0.36	0.32
P3			0.4	0.328
P4				0.2848
For allele frequency				
Mean ( 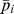 )	0.4	0.4	0.4	
Variance-covariance ( 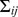 )				
P1	0.1	0.01	0.04	
P2		0.1	0.09	
P3			0.1	
Admixture proportion				
P4	0.2	0.6	0.2	
Eigenvalue	3599.06	204.80	0.32	−0.00
Eigenvector				
P1	−0.82	−0.10	−0.17	0.50
P2	0.50	−0.48	−0.58	0.50
P3	0.23	0.86	−0.22	0.50
P4	0.18	−0.13	0.77	0.50

When an additional population that is not related to admixture is included in PCA, in the three-dimensional space spanned by the first three eigenvectors, the representative point of the admixed population will still be located on the plane formed by the representative points of the three parental populations and will divide the triangle according to the admixture proportions by Equations (21), (22) and (23). Note that the triangle formed by the representative points of the three parental populations does not necessarily lie in a plane formed by any two of the first three eigenvectors. This inclined triangle formed by the admixed population and the parental populations in a three-dimensional space is reminiscent of the slanted segment for a two-way admixed population and the parental populations in a two-dimensional space. We confirmed this pattern by numerically solving the reduced eigenequation (1) for 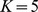 with various different values of variance-covariance parameters and admixture parameters. An example is given in Figure S4.

**Figure 6:**
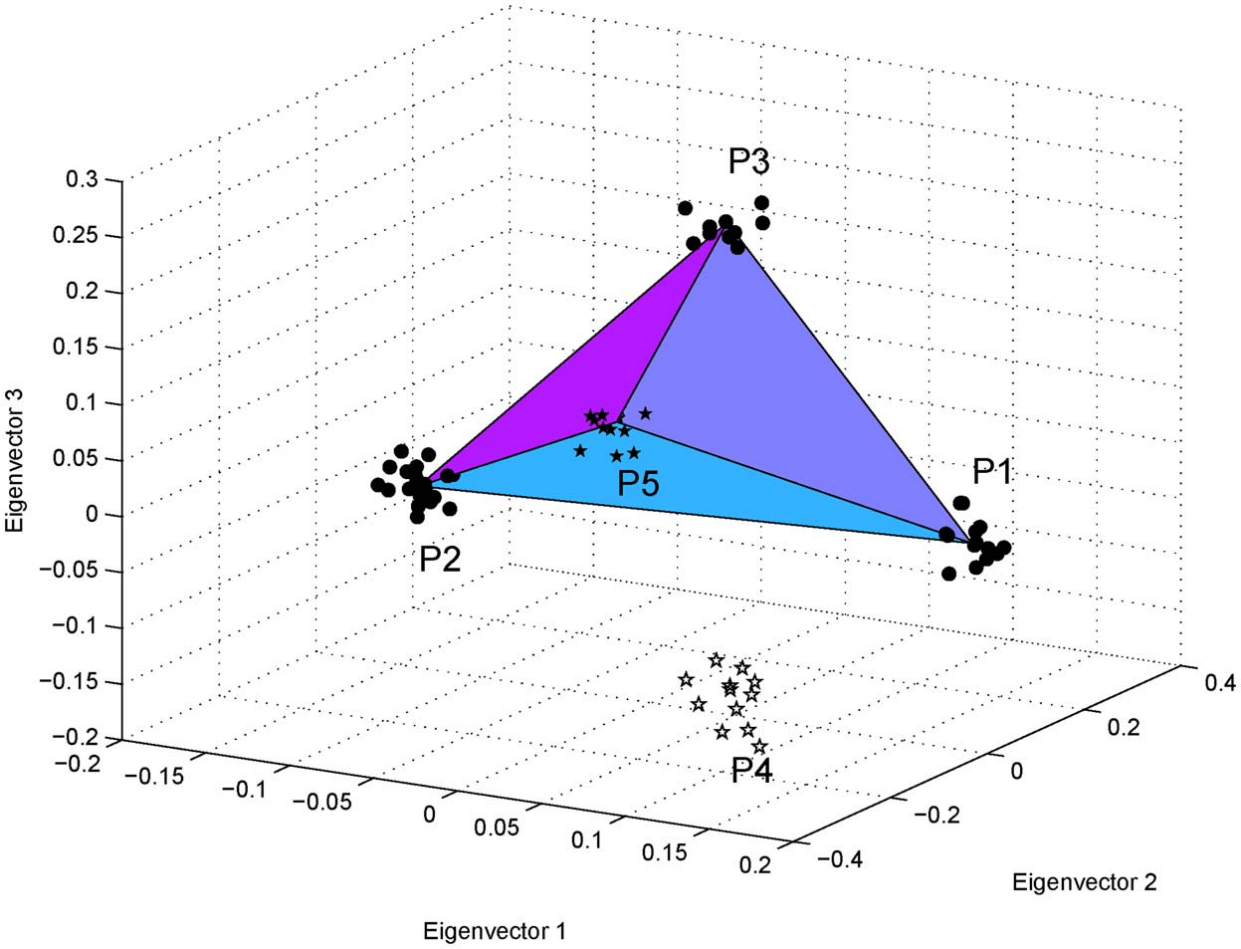
Simulation of a genetically homogeneous admixed population with an additional population: three-way admixture. The first three eigenvectors are shown for a simulated data set with five populations. P5, with sample size 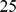, was an admixed population of P1, P2, and P3 with admixture proportions 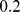: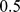: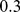. P4 was an additional population. P1, P2, P3, and P4 were simulated using 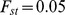, with sample sizes 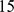, 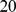, 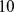, and 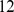, respectively. In the three-dimensional space, the samples from P5 were found to cluster around a point inside the triangle formed by the centroids of the three parental populations, and they divided the triangle into three small triangles, the ratio of the areas of which was approximately equal to the corresponding ratio of the simulating admixture proportions. Population P4 was included to introduce an additional dimension of variation, so that the admixed population and the parental populations formed an inclined triangle.

In Figure 6, we show an example of simulations for a three-way admixture. Here, P5 was an admixed population, with sample size 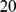, of P1, P2, and P3, with admixture proportions of 0.2∶0.5∶0.3 and sample sizes of 15, 25, and 10, respectively. P4 was an additional population with sample size 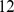. P1, P2, P3, and P4 were simulated using 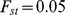 (see Methods). The centroids of all five populations were calculated by solving the reduced eigenequation (1) with variance-covariance parameters estimated using the method given in [6]. We can see that the samples from P5 lie approximately in the plane formed by the centroids of the three parental populations; the distance from the centroid of the P5 samples to the plane is 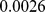, much smaller than the dimensions of the triangle (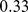, 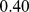, and 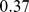). The admixture proportions were estimated using Equations (21), (22) and (23) as 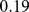: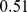: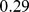, very close to the simulating values. In contrast, the performance of STRUCTURE was poor in the case of three-way admixture; the admixture proportions estimated using STRUCTURE were 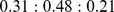.

**Figure 7:**
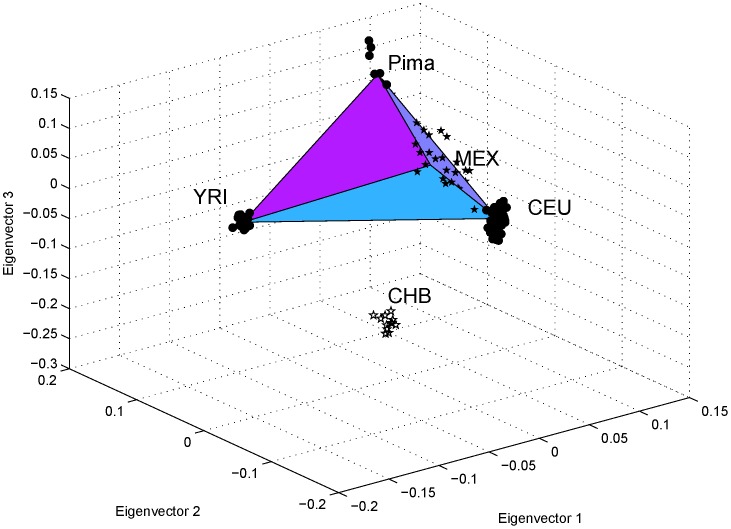
An example of three-way admixture from the HapMap and HGDP samples. The first three eigenvectors are shown for pooled data of the HapMap populations CEU, CHB, MEX, and YRI and the HGDP population Pima. Samples from MEX were found to be distributed around the inclined triangular plane formed by the clusters of CEU, Pima, and YRI, and most of them were inside the triangle. CEU, Pima, and YRI served as the proxy parental populations of the MEX population. CHB was included to introduce an additional dimension of variation, so that the three-way admixture-related populations formed an inclined triangle.

Figure 7 shows the results for four HapMap populations, CEU, CHB, MEX, and YRI, and one HGDP population, Pima, as a real example of three-way admixture. PCA was performed for the intersection of the HapMap III and HGDP marker sets. Here, CEU, Pima, and YRI were used to represent the European, Native American and African ancestries, respectively, of the MEX population. CHB was included in the analysis to provide an additional axis of variation. The MEX samples were found to lie approximately in the plane formed by the centroids of the CEU, Pima, and YRI samples in the three-dimensional space spanned by the first three eigenvectors (Figure 7): The distance from the centroid of the MEX samples to the plane is 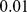, which is small compared with the dimensions of the triangle (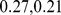, and 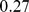). The results confirmed that the MEX population is a genetically recent admixture with an average of 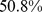 of European, 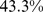 of Native American, and 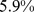 of African ancestry, as calculated using Equations (21), (22) and (23) with P4 representing the centroid of the MEX samples. The individual admixture proportions of the MEX samples were calculated also using Equations (21), (22) and (23) and are listed in Table S2. Also shown in Table S2 were the results of STRUCTURE. The average proportions estimated by STRUCTURE were 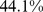, 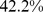, and 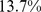, for European, Native American and African ancestry, respectively. The Spearman correlation coefficients between our PCA method and STRUCTURE for these three admixture proportions were 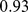, 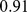, and 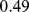, respectively.

### Admixture with an Arbitrary Number of Ancestral Populations

The relationship between the geometric properties of the eigenvector-plot and the admixture proportions can be extended to the general case of multiple parental populations. For example, if there are four parental populations, the representative point of the admixed population will be located inside a tetrahedron with the representative points of the four parental populations on the four vertices, and will divide the tetrahedron into four small tetrahedra, the ratio of the volumes of which will be the corresponding ratio of the admixture proportions.

In general, if population 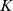 is an admixture of 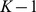 other populations, we have the eigenvectors of the reduced eigenequation (7) listed in the columns as follows:
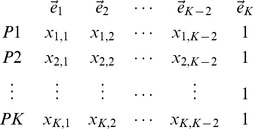
(24)where the 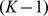
th eigenvector is discarded because it corresponds to the eigenvalue of zero in the asymptotic limit because of admixture. In the case of two-way admixture, this eigenvector is given by Equation (20). The trivial eigenvector, 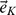, which reflects the mean adjustment (2), is listed for a reason that will soon be clear. For the admixture proportion of the 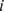
th parental population,
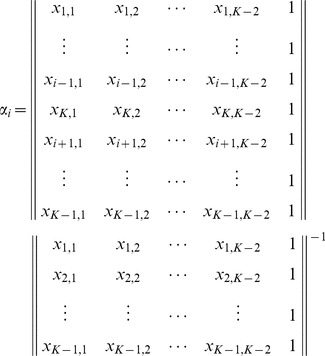
(25)for 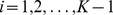, where 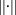 denotes the absolute value of the determinant of a matrix. The matrix in the denominator is simply the first 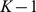 rows of the matrix given in Equation (24), and the matrix in the numerator is obtained by replacing the 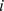
th row of the matrix in the denominator with the last row of the matrix in Equation (24).

It can easily be seen that the expressions for the 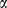
s are the solutions of the following equations:
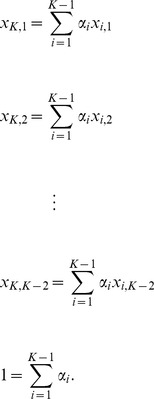
(26)


This implies that the component corresponding to the admixed population in each main eigenvector consists of the components of the parental populations in the same proportions as the admixture proportions. In other words, the position of an admixed sample is given by the weighted sum of the positions of the parental populations, a statement similar to what is proposed in [7] for conventional PCA with markers as features. The weights are simply the corresponding admixture proportions.

## Discussion

The study of population admixture can be dated back to the 1970s [11], although there were few markers that can be used to estimate admixture proportions. We have extended our previously developed theoretical formulation of PCA to the general case where some of the populations may be admixtures of others. Our generalized formulation can be used to theoretically predict the pattern of the eigenvector-plot from PCA using pre-specified parameters reflecting the population structures, relationships and admixture proportions. For real data analysis in population genetics and genetic epidemiology, our formulation can provide theoretical guidance on how to properly infer population structure and relationships, how to identify admixed individuals, and how to distinguish admixture distributions from within-population variations. Based on theoretical investigations using our formulation, we have justified the diagnostic of two-way admixture, and more importantly, proposed diagnostics of multi-way admixture, which can also be used to estimate admixture proportions.

In a majority of literature on PCA, as a tool to detect population structure, individuals are treated as samples and markers as features in a natural way. In our formulation, we adopt the other statistical viewpoint: individuals sampled from various populations are treated as features and the markers as “samples” or realizations. This is why the parameters reflecting the population properties and admixture proportions can be defined *a priori*, and thus can be directly related to the theoretical pattern of the eigenvector-plot. Although conceptually different, the eigen-solutions for these two approaches are found to be almost identical up to a scale constant (data not shown). Specifically, the pattern of the scatter plot of PCs is the same as that of the eigenvector-plot in our formulation. Therefore, the diagnostics of admixture and the method of estimating admixture proportions obtained here from our formulation should apply to the PC scatter plot (obtained using, e.g., EIGENSTRAT) widely used in the literature.

In our theoretical formulation of PCA, an important issue is that values of the elements of the variance-covariance matrix depend on the coding of alleles, which is not specified. Specifically, the covariances will be largest if the alleles of markers are randomly labelled, and smallest if the major (or minor) allele is always chosen as the variant allele. Simulation studies showed that the pattern of eigenvector-plot is not influenced by the coding of alleles (data not shown). However, if the variance-covariance parameters are used to measure the population structures and relationships, the coding of alleles should be prespecified for all markers.

For the estimation of admixture proportions, we have compared our results obtained using PCA with those obtained using STRUCTURE [12], and found that they were highly correlated for both simulated data and real data. Although it seems that our PCA-based method outperformed STRUCTURE for the simulated data sets, it should be noted that a fair comparison of our method with STRUCTURE or other existing methods requires comprehensive and extensive simulations for various scenarios, and thus should be left for a future investigation.

In the present work, we have focused on the overall ancestry proportions of admixed populations by performing PCA on a genome- or chromosome-wide scale. Estimation of the local ancestry of admixed samples is critical for the admixture mapping approach. The ancestral structure of an admixed genome may also yield new insights into the evolutionary history of human populations. Recently, a genome scan approach based on PCA and wavelet transform analysis has been proposed to estimate the time of admixture [13]. In a separate study, we have proposed a method to detect chromosomal inversions by performing PCA locally (Ma and Amos, to be published). Our method was based on the fact that suppression of recombination in inversion heterozygotes due to the loss of unbalanced gametes creates a local population substructure: two distinct “populations” of inversion homozygotes of different orientations and their 1∶1 admixture, namely the inversion heterozygotes. Our analysis with the HapMap data shows that locally performed PCA can identify this substructure on the scale of 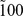 kb. We expect that the proposed diagnostics of admixture here would prove useful for determining the local admixture structure of individual admixed genomes.

In our previous work [6], we have analytically shown and numerically demonstrated that simply removing the first few PCs from the allele counts is equivalent to subtracting the population group means, a simple method to correct for population stratification. Although including admixed samples whose parental populations are already present in the analysis does not add to the number of axes of variation, as shown here in the case of two-way admixture, not all markers follow the global admixture proportion. It would be interesting to figure out how many PCs should be removed for correcting population stratification and whether it is still equivalent to removing the group means in the case where admixed samples are included in the analysis. Answers to these questions would be important for genetic association tests using admixed populations, especially for tests of gene-gene and gene-environment interaction using case-only approaches.

## Methods

### Simulations of Population Structure

We used the same method as in [14], [2], and [6] to simulate genotype data for non-admixed populations with pre-specified values of 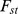. Briefly, the method of simulation is based on the Balding-Nichols model [15]. We first generated the overall ancestral allele frequencies for all markers separately from the uniform distribution on 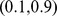. For each population, say, population 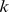, the allele frequencies were then drawn from a beta distribution with parameters 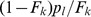 and 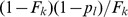, where 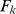 was the value of 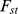 for this population and 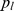 was the overall ancestral allele frequency for locus 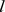. The generated allele frequencies were then used to generate the genotypes for each of the individuals in the population by assuming Hardy-Weinberg equilibrium.

### Simulations of Admixture

Two or three populations generated in this way can be chosen as parental populations, and an admixed population was simulated using the following approach. To simulate a well-admixed population, we draw an allele frequency for a marker for each individual in the admixed population from the allele frequencies of the parental populations according to the pre-specified, common admixture proportions. For example, if there are three parental populations with allele frequencies, 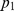, 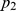, and 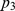, and the admixture proportions are 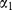: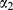: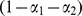, the allele frequency of the admixed population may be 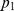, 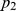, or 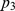, with probabilities 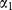, 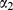, or 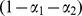, respectively. For a recently admixed population, the allele frequency was still generated from those of the parental populations, but according to different admixture proportions drawn from a beta distribution with parameters 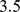 and 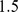.

### Datasets Used

Unphased genotype data for the ASW, MEX, TSI, and YRI populations from the International HapMap Project (HapMap III release 2) and for the HGDP population Pima. PCA of simulated data, HapMap data, and HGDP data was carried out using the R function eigen (www.r-project.org).

### Comparison with STRUCTURE

Estimation of admixture proportions using PCA was compared with that of STRUCTURE. For all STRUCTURE runs, we ran with a burn-in of 10,000 iterations with 40,000 follow-on iterations. The admixture model was used, and the population ID for each ancestral populations were used as prior information. The posterior mean estimates of ancestry proportions were obtained for each individual in the admixed population. We used the first 300 AIMs for the populations involved in the analysis. Similar results were obtained when more AIMs (e.g. 600 SNPs) were used to run STRUCTURE for the examples we were interested here. To determine the AIMs for the samples in each analysis, we rank the SNPs according to their capability of distinguishing populations using the following approach. Suppose there are 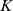 distinct populations (excluding any admixed populations of them). We will need 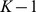 eigenvectors to distinguish them. For each of these eigenvectors, 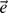, we first calculate the projections of all markers:
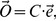
(27)where 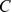 is the data matrix. Then we sort the elements of 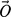 according to their absolute values. The markers with large values of 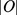 will be chosen as the most informative markers for the populations addressed by the iegenvector 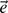.

Programs and scripts used in this work are available from the authors upon request.

## Supporting Information

Figure S1
**Theoretical prediction of PCA: two-way admixture.** The first three eigenvectors of four hypothetical populations, P1, P2, P3, and P4, were calculated from the reduced eigenequation (1). Populations P1, P2, and P4 were defined by the following parameters: allele frequency mean, 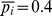 for 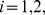 and 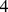; variance-covariance parameters, 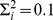 for 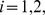 and 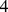, 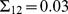, and 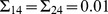. P3 was an admixture of P1 and P2 with admixture proportions 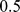: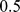. Samples sizes for P1, P2, P3, and P4 were 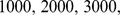 and 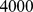, respectively. In the plane spanned by the first two eigenvectors (left panel), P3 was located on the line segment connecting P1 and P2 and divided the line segment in the ratio of the admixture proportions: 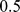: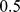. Because there were three independent populations (P1, P2, and P4), two eigenvectors were needed to address the population-level variations. Along the third eigenvector (right panel), P3 was located outside the interval of P1 and P2, indicating that the third PC addresses within-population variations.(EPS)

Figure S2 **Performance comparison of two approaches of estimating individual admixture proportions: PCA vs. STRUCTURE.** We show the deviations of the estimated individual admixture proportions, using the proposed PCA-based method and STRUCTURE, from the simulating values for the simulated data shown in Figure 3.
(EPS)

Figure S3
**Theoretical prediction of PCA: three-way admixture.** The first four eigenvectors of five hypothetical populations, P1, P2, P3, P4, and P5, were calculated from the reduced eigenequation (1). Populations P1, P2, and P3 were defined by the following parameters: allele frequency mean, 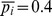 for 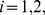 and 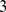; variance-covariance parameters, 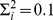 for 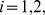 and 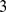, 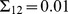, 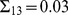, and 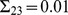. P4 and P5 were admixed populations of P1, P2, and P3 with admixture proportions 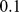: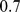: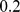 and 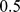: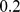: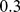, respectively. In the two-dimensional space spanned by the first two eigenvectors, P4 and P5 were inside the triangle formed by P1,P2, and P3 and divided the triangle into three small triangles with areas in the ratio of the admixture proportions: 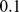: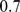: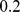 and 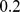: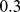: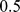, respectively. Because there were three independent populations, the third eigenvector did not address population-level variation (right bottom panel). It should be noted that the distances between P4 and P1 and between P4 were almost identical (0.083 and 0.080, respectively), although the admixture proportion of P3 (0.2) is twice that of P1 (0.1). This is an illustration that it is the areas, not the lengths, that should be used to measure the admixture proportions in three-way admixture.(EPS)

Figure S4
**Theoretical prediction of PCA: three-way admixture with an additional population.** The first four eigenvectors of five hypothetical populations, P1, P2, P3, P4, and P5, were calculated from the reduced eigenequation (1). Populations P1, P2, P3, and P5 were defined by the following parameters: allele frequency mean, 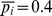 for 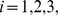 and 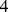; variance-covariance parameters, 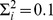 for 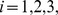 and 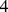, 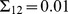, 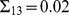, 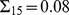, 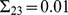, 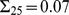, and 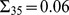. P4 was an admixture of P1, P2, and P3 with admixture proportions, 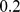: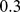: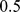. In each of the two-dimensional spaces spanned by any two of the first three eigenvectors, P4 was located inside the triangle formed by P1, P2, and P3 and divided the triangle into three small triangles with areas in the ratio of the admixture proportions: 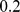: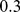: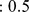. This implies that in the three-dimensional space spanned by the first three eigenvectors, P4 was on the same plane defined by P1, P2, and P3 and divided the triangle in the same ratio. Because there were four independent populations, the fourth eigenvector did not address population-level variation (right bottom panel).(EPS)

Table S1 Estimated admixture proportions of the ASW samples.
(XLS)

Table S2 Estimated admixture proportions of the MEX samples.
(XLS)

Text S1 The variance-covariance parameters of variant allele count in terms of those of allele frequency.
(PDF)

Text S2 Inferring admixture proportions from the asymptotic pattern of the eigenvector-plot: two-way admixture.
(PDF)

Text S3 Individual-level inference of admixture proportions.
(PDF)
