# Supplementary material for: Principal Components Analysis of Population Admixture
Source: PLoS One. 2012 Jul 9;7(7):e40115. doi: 10.1371/journal.pone.0040115 (PMC3392282; doi:10.1371/journal.pone.0040115)
Supplement: Text S1 — The variance-covariance parameters of variant allele count in terms of those of allele frequency. (PDF) [file pone.0040115.s007.pdf]

Principal components analysis of population admixture

Jianzhong Ma*, Christopher I. Amos
Department of Genetics, The University of Texas MD Anderson Cancer Center, Houston, Texas, USA
* **E-mail: jzma@mdanderson.org**

Text S1: The variance-covariance parameters of variant allele count in terms of those of allele frequency

Suppose that there are five distinct populations: P1, P2, P3, P4, and P5. P3 and P4 are admixtures of P1 and P2 with proportions α:$(1-\alpha)$ and β:$(1-\beta)$, respectively. For a random marker, the variant allele frequencies of P1, P2, and P5, p_1, p_2 and p_5, are random variables with a variance-covariance matrix

$$V_F = \begin{pmatrix} \Sigma_1^2 & \Sigma_{12} & \Sigma_{15} \\ \Sigma_{12} & \Sigma_2^2 & \Sigma_{25} \\ \Sigma_{15} & \Sigma_{25} & \Sigma_5^2 \end{pmatrix}.$$

For P3, consider the probability that the paternal allele is the variant allele, given the allele frequencies of P1 and P2

$$\begin{aligned} P(a_3^{\text{pat}} = 1|p_1, p_2) &= P(a_3^{\text{pat}} = 1|\text{ancestry=P1}, p_1, p_2)P(\text{ancestry=P1}|p_1, p_2) \\ &\quad + P(a_3^{\text{pat}} = 1|\text{ancestry=P2}, p_1, p_2)P(\text{ancestry=P2}|p_1, p_2) \\ &= p_1\alpha + p_2(1-\alpha). \end{aligned}$$

The same is true for the maternal allele. Assuming random mating, we have the allele frequency of P3

$$p_3 = p_1\alpha + p_2(1-\alpha)$$

and similarly of P4

$$p_4 = p_1\beta + p_2(1-\beta).$$

As in Text S1 of [1], we can show that

$$\begin{aligned} P(C_1) &= \int \mathrm{d}p_1 P(C_1|p_1)P(p_1) \\ P(C_2) &= \int \mathrm{d}p_2 P(C_2|p_2)P(p_2) \\ P(C_5) &= \int \mathrm{d}p_5 P(C_5|p_5)P(p_5) \\ P(C_3) &= \int \mathrm{d}p_1\mathrm{d}p_2 P(p_1, p_2)P(C_3|p_1, p_2) \\ P(C_4) &= \int \mathrm{d}p_1\mathrm{d}p_2 P(p_1, p_2)P(C_4|p_1, p_2) \\ P(C_1, C_2) &= \int \mathrm{d}p_1\mathrm{d}p_2 P(p_1, p_2)P(C_1|p_1)P(C_2|p_2) \\ P(C_1, C_5) &= \int \mathrm{d}p_1\mathrm{d}p_5 P(p_1, p_5)P(C_1|p_1)P(C_5|p_5) \\ P(C_2, C_5) &= \int \mathrm{d}p_2\mathrm{d}p_5 P(p_2, p_5)P(C_2|p_2)P(C_5|p_5) \\ P(C_1, C_3) &= \int \mathrm{d}p_1\mathrm{d}p_2 P(p_1, p_2)P(C_1|p_1)P(C_3|p_1, p_2) \\ P(C_2, C_3) &= \int \mathrm{d}p_1\mathrm{d}p_2 P(p_1, p_2)P(C_2|p_2)P(C_3|p_1, p_2) \end{aligned}$$

$$
\begin{aligned}
P(C_1, C_4) &= \int \mathrm{d}p_1 \mathrm{d}p_2 P(p_1, p_2) P(C_1|p_1) P(C_4|p_1, p_2) \\
P(C_2, C_4) &= \int \mathrm{d}p_1 \mathrm{d}p_2 P(p_1, p_2) P(C_2|p_2) P(C_4|p_1, p_2) \\
P(C_3, C_4) &= \int \mathrm{d}p_1 \mathrm{d}p_2 P(p_1, p_2) P(C_3|p_1, p_2) P(C_4|p_1, p_2) \\
P(C_3, C_5) &= \int \mathrm{d}p_1 \mathrm{d}p_2 \mathrm{d}p_5 P(p_1, p_2, p_5) P(C_3|p_1, p_2) P(C_5|p_5) \\
P(C_4, C_5) &= \int \mathrm{d}p_1 \mathrm{d}p_2 \mathrm{d}p_5 P(p_1, p_2, p_5) P(C_4|p_1, p_2) P(C_5|p_5),
\end{aligned}
$$

where C_1, C_2, C_3, C_4, and C_5 are the variant allele counts for an individual in P1, P2, P3, P4, and P5, respectively. As an example, here we prove the expression for $P(C_3, C_4)$.

$$
\begin{aligned}
P(C_3, C_4) &= \int \mathrm{d}p_1 \mathrm{d}p_2 \mathrm{d}p_5 \prod_{i(\neq 3,4)} \sum_{C_i} P(C_1, C_2, \cdots | p_1, p_2, p_5) P(p_1, p_2, p_5) \\
&= \int \mathrm{d}p_1 \mathrm{d}p_2 P(C_3|p_1, p_2) P(C_4|p_1, p_2) \int \mathrm{d}p_5 P(p_1, p_2, p_5) \prod_{i(\neq 3,4)} \sum_{C_i} P(C_i|p_1, p_2, p_5) \\
&= \int \mathrm{d}p_1 \mathrm{d}p_2 P(p_1, p_2) P(C_3|p_1, p_2) P(C_4|p_1, p_2).
\end{aligned}
$$

Using these marginal probabilities and Hardy-Weinberg proportion, we can obtain the variance-covariance parameters as follows:

$$
\begin{aligned}
\sigma_1^2 &= 2\Sigma_1^2 + 2\overline{p_1}(1 - \overline{p_1}) \\
\sigma_2^2 &= 2\Sigma_2^2 + 2\overline{p_2}(1 - \overline{p_2}) \\
\sigma_5^2 &= 2\Sigma_5^2 + 2\overline{p_5}(1 - \overline{p_5}) \\
\sigma_3^2 &= 2\alpha^2\Sigma_1^2 + 2(1-\alpha)^2\Sigma_2^2 + 4\alpha(1-\alpha)\Sigma_{12} + 2[\alpha\overline{p_1} + (1-\alpha)\overline{p_2}][1 - \alpha\overline{p_1} + (1-\alpha)\overline{p_2}] \\
\sigma_4^2 &= 2\beta^2\Sigma_1^2 + 2(1-\beta)^2\Sigma_2^2 + 4\beta(1-\beta)\Sigma_{12} + 2[\beta\overline{p_1} + (1-\beta)\overline{p_2}][1 - \beta\overline{p_1} + (1-\beta)\overline{p_2}] \\
\sigma_{11} &= 4\Sigma_1^2 \\
\sigma_{22} &= 4\Sigma_2^2 \\
\sigma_{55} &= 4\Sigma_5^2 \\
\sigma_{33} &= 4\alpha^2\Sigma_1^2 + 4(1-\alpha)^2\Sigma_2^2 + 8\alpha(1-\alpha)\Sigma_{12} \\
\sigma_{44} &= 4\beta^2\Sigma_1^2 + 4(1-\beta)^2\Sigma_2^2 + 8\beta(1-\beta)\Sigma_{12} \\
\sigma_{12} &= 4\Sigma_{12} \\
\sigma_{15} &= 4\Sigma_{15} \\
\sigma_{25} &= 4\Sigma_{25} \\
\sigma_{13} &= 4\alpha\Sigma_1^2 + 4(1-\alpha)\Sigma_{12} \\
\sigma_{23} &= 4(1-\alpha)\Sigma_2^2 + 4\alpha\Sigma_{12} \\
\sigma_{14} &= 4\beta\Sigma_1^2 + 4(1-\beta)\Sigma_{12} \\
\sigma_{24} &= 4(1-\beta)\Sigma_2^2 + 4\beta\Sigma_{12} \\
\sigma_{35} &= 4\alpha\Sigma_{15} + 4(1-\alpha)\Sigma_{25} \\
\sigma_{45} &= 4\beta\Sigma_{15} + 4(1-\beta)\Sigma_{25} \\
\sigma_{34} &= 4\alpha\beta\Sigma_1^2 + 4(1-\alpha)(1-\beta)\Sigma_2^2 + 4(\alpha + \beta - 2\alpha\beta)\Sigma_{12}.
\end{aligned}
$$

Here, as an example, we prove the expression for σ_{34}. Using Hardy-Weinberg proportion,

$$P(C_3|p_1,p_2) = \begin{cases} (1-p_3)^2 & (C_3=0) \\ 2p_3(1-p_3) & (C_3=1) \\ p_3^2 & (C_3=2) \end{cases}$$

and a similar equation for $P(C_4|p_1,p_2)$, we have

$$\begin{aligned} \overline{C_3} &= \int \mathrm{d}p_1p_2 \sum_{C_3} P(p_1,p_2)C_3P(C_3|p_1,p_2) = 2[\alpha\overline{p_1} + (1-\alpha)\overline{p_2}] \\ \overline{C_4} &= \int \mathrm{d}p_1p_2 \sum_{C_3} P(p_1,p_2)C_3P(C_3|p_1,p_2) = 2[\beta\overline{p_1} + (1-\beta)\overline{p_2}] \end{aligned}$$

and

$$\begin{aligned} \overline{C_3C_4} &= \int \mathrm{d}p_1p_2P(p_1,p_2)\sum_{C_3} C_3P(C_3|p_1,p_2)\sum_{C_4} C_4P(C_4|p_1,p_2) \\ &= \int \mathrm{d}p_1p_2P(p_1,p_2)2p_32p_4 \\ &= 4\alpha\beta\overline{p_1^2} + 4(1-\alpha)(1-\beta)\overline{p_2^2} + 4(\alpha+\beta-2\alpha\beta)\overline{p_1p_2}, \end{aligned}$$

and hence

$$\sigma_{34} \equiv \overline{C_3C_4} - \overline{C_3}\overline{C_4} = 4\alpha\beta\Sigma_1^2 + 4(1-\alpha)(1-\beta)\Sigma_2^2 + 4(\alpha+\beta-2\alpha\beta)\Sigma_{12}.$$

These results can be generalized to the case where there are multiple ancestral populations. Suppose that we have six populations, P1, P2, P3, P4, P5, and P6, among which P4 and P5 are admixed populations of P1, P2, and P3 with proportions α_1:α_2:$(1-\alpha_1-\alpha_2)$ and β_1:β_2:$(1-\beta_1-\beta_2)$, respectively. The expressions that are different from those in the two-ancestry case are as follows:

$$\begin{aligned} \sigma_4^2 &= 2\alpha_1^2\Sigma_1^2 + 2\alpha_2^2\Sigma_2^2 + 2(1-\alpha_1-\alpha_2)^2\Sigma_3^2 + 4\alpha_1\alpha_2\Sigma_{12} \\ &\quad +4\alpha_1(1-\alpha_1-\alpha_2)\Sigma_{13} + 4\alpha_2(1-\alpha_1-\alpha_2)\Sigma_{23} + 2\overline{p_4}(1-\overline{p_4}) \\ \sigma_{44} &= 4\alpha_1^2\Sigma_1^2 + 4\alpha_2^2\Sigma_2^2 + 4(1-\alpha_1-\alpha_2)^2\Sigma_3^2 \\ &\quad +8\alpha_1\alpha_2\sigma_{12} + 8\alpha_1(1-\alpha_1-\alpha_2)\Sigma_{13} + 8\alpha_2(1-\alpha_1-\alpha_2)\Sigma_{23} \\ \sigma_{14} &= 4\alpha_1\Sigma_1^2 + 4\alpha_2\Sigma_{12} + 4(1-\alpha_1-\alpha_2)\Sigma_{13} \\ \sigma_{24} &= 4\alpha_2\Sigma_2^2 + 4\alpha_1\Sigma_{12} + 4(1-\alpha_1-\alpha_2)\Sigma_{23} \\ \sigma_{34} &= 4(1-\alpha_1-\alpha_2)\Sigma_3^2 + 4\alpha_1\Sigma_{13} + 4\alpha_2\Sigma_{23} \\ \sigma_{45} &= 4\alpha_1\beta_1\Sigma_1^2 + 4\alpha_2\beta_2\Sigma_2^2 + 4(1-\alpha_1-\alpha_2)(1-\beta_1-\beta_2)\Sigma_3^3 + 4(\alpha_1\beta_2+\alpha_2\beta_1)\Sigma_{12} \\ &\quad +4(\alpha_1+\beta_1-2\alpha_1\beta_1-\alpha_1\beta_2-\alpha_2\beta_1)\Sigma_{13} + 4(\alpha_2+\beta_2-2\alpha_2\beta_2-\alpha_1\beta_2-\alpha_2\beta_1)\Sigma_{23} \\ \sigma_{46} &= 4\alpha_1\Sigma_{15} + 4\alpha_2\Sigma_{26} + 4(1-\alpha_1-\alpha_2)\Sigma_{36}. \end{aligned}$$

The expressions for σ_5^2, σ_{55}, σ_{15}, σ_{25}, σ_{35}, and σ_{56} are the same as those for σ_4^2, σ_{44}, σ_{14}, σ_{24}, σ_{34} and σ_{46}, respectively, except that the αs are replaced by the corresponding βs.

References

1. Ma J, Amos C (2010) Theoretical formulation of principal components analysis to detect and correct for population stratification. PLoS ONE 5: e12510.
