# Supplementary material for: Principal Components Analysis of Population Admixture
Source: PLoS One. 2012 Jul 9;7(7):e40115. doi: 10.1371/journal.pone.0040115 (PMC3392282; doi:10.1371/journal.pone.0040115)
Supplement: Text S2 — Inferring admixture proportions from the asymptotic pattern of the eigenvector-plot: two-way admixture. (PDF) [file pone.0040115.s008.pdf]

Principal components analysis of population admixture

Jianzhong Ma*, Christopher I. Amos
Department of Genetics, The University of Texas MD Anderson Cancer Center, Houston, Texas, USA
* **E-mail: jzma@mdanderson.org**

Text S2: Inferring admixture proportions from the asymptotic pattern of the eigenvector-plot: two-way admixture

If there are three populations, $K = 3$, the reduced eigenequation

$$\begin{pmatrix} -(N_2\sigma'_{12} + N_3\sigma'_{13}) & N_2\sigma'_{12} & N_3\sigma'_{13} \\ N_1\sigma'_{12} & -(N_1\sigma'_{12} + N_3\sigma'_{23}) & N_3\sigma'_{23} \\ N_1\sigma'_{13} & N_2\sigma'_{23} & -(N_1\sigma'_{13} + N_2\sigma'_{23}) \end{pmatrix} \begin{pmatrix} x_1 \\ x_2 \\ x_3 \end{pmatrix} = \lambda \begin{pmatrix} x_1 \\ x_2 \\ x_3 \end{pmatrix}$$

can be analytically solved. In addition to the trivial solution

$$\lambda_1 = 0, \quad (x_1, x_2, x_3) = \frac{1}{\sqrt{N_1 + N_2 + N_3}}(1, 1, 1),$$

there are two more solutions, of which the eigenvalues are the solutions of

$$\begin{aligned} \lambda^2 + \lambda\left[(N_1 + N_2)\sigma'_{12} + (N_1 + N_3)\sigma'_{13} + (N_2 + N_3)\sigma'_{23}\right] + \\ \left[N_1\sigma'_{12}\sigma'_{13} + N_2\sigma'_{12}\sigma'_{23} + N_3\sigma'_{13}\sigma'_{23}\right](N_1 + N_2 + N_3) &= 0. \end{aligned}$$

In the asymptotic limit ($N \to \infty$), the reduced eigenequation is

$$N \begin{pmatrix} -(n_1\hat{\sigma}_{12} + n_2\hat{\sigma}_{13}) & n_2\hat{\sigma}_{12} & n_3\hat{\sigma}_{13} \\ n_1\hat{\sigma}_{12} & -(n_1\hat{\sigma}_{12} + n_2\hat{\sigma}_{23}) & n_3\hat{\sigma}_{23} \\ n_1\hat{\sigma}_{13} & n_2\hat{\sigma}_{23} & -(n_1\hat{\sigma}_{13} + n_2\hat{\sigma}_{23}) \end{pmatrix} \begin{pmatrix} x_1 \\ x_2 \\ x_3 \end{pmatrix} = \lambda^* \begin{pmatrix} x_1 \\ x_2 \\ x_3 \end{pmatrix}.$$

The two eigenvalues then are determined by

$$\begin{aligned} \left(\frac{\lambda^*}{N}\right)^2 + \left(\frac{\lambda^*}{N}\right)\left[(n_1 + n_2)\hat{\sigma}_{12} + (n_1 + n_3)\hat{\sigma}_{13} + (n_2 + n_3)\hat{\sigma}_{23}\right] + \\ \left[n_1\hat{\sigma}_{12}\hat{\sigma}_{13} + n_2\hat{\sigma}_{12}\hat{\sigma}_{23} + n_3\hat{\sigma}_{13}\hat{\sigma}_{23}\right] = 0. \end{aligned}$$

The coefficients of this equation is independent of N, and hence its solutions are linear functions of N.

Now suppose that population P3 is an admixed population of the other two populations, P1 and P2, with admixture proportions α:$(1 - \alpha)$. Using the expressions derived in Text S1, we have, after tedious algebra,

$$\begin{aligned} \hat{\sigma}_{12} &= -4[n_1 + \alpha n_3][n_2 + (1 - \alpha)n_3]\left[\Sigma_1^2 + \Sigma_2^2 - 2\Sigma_{12}\right] \\ \hat{\sigma}_{13} &= 4[n_2 + (1 - \alpha)n_3][\alpha n_2 - (1 - \alpha)n_1]\left[\Sigma_1^2 + \Sigma_2^2 - 2\Sigma_{12}\right] \\ \hat{\sigma}_{23} &= -4[n_1 + \alpha n_3][\alpha n_2 - (1 - \alpha)n_1]\left[\Sigma_1^2 + \Sigma_2^2 - 2\Sigma_{12}\right]. \end{aligned}$$

These expressions lead to an important sum-rule

$$n_1\hat{\sigma}_{12}\hat{\sigma}_{13} + n_2\hat{\sigma}_{12}\hat{\sigma}_{23} + n_3\hat{\sigma}_{13}\hat{\sigma}_{23} = 0.$$

The two asymptotic eigenvalues are now

$$\begin{aligned} \lambda_1^* &= -N[(n_1 + n_2)\hat{\sigma}_{12} + (n_1 + n_3)\hat{\sigma}_{13} + (n_2 + n_3)\hat{\sigma}_{23}] \\ \lambda_2^* &= 0. \end{aligned}$$

Substituting the non-zero eigenvalue, λ_1^*, into the eigenequation, we find that the eigenvector satisfies

$$x_1 a = x_2 b = x_3 c,$$

where

$$\begin{aligned} a &= -\lambda_1^* \hat{\sigma}_{23} \\ b &= -\lambda_1^* \hat{\sigma}_{13} \\ c &= -\lambda_1^* \hat{\sigma}_{12}. \end{aligned}$$

Hence, up to a normalization constant, the eigenvector is

$$\begin{pmatrix} x_1 \\ x_2 \\ x_3 \end{pmatrix} = \begin{pmatrix} -[n_2 + (1-\alpha) n_3] \\ [n_1 + \alpha n_3] \\ -[\alpha n_2 - (1-\alpha) n_1] \end{pmatrix}.$$

Finally, we have

$$\begin{aligned} \frac{x_3 - x_1}{x_2 - x_1} &= 1 - \alpha \\ \frac{x_2 - x_3}{x_2 - x_1} &= \alpha. \end{aligned}$$

For the other eigenvalue, $\lambda_2^* = 0$, the corresponding eigenvector, $(X_1, X_2, X_3)^T$, can be obtained as follows. It must be orthogonal with $(1, 1, 1)$, the first eigenvector:

$$n_1 X_1 + n_2 X_2 + n_3 X_3 = 0,$$

and with the one corresponding to λ_1^*

$$n_1 x_1 X_1 + n_2 x_2 X_2 + n_3 x_3 X_3 = 0.$$

This leads to the expression of the eigenvector:

$$\begin{pmatrix} X_1 \\ X_2 \\ X_3 \end{pmatrix} = \begin{pmatrix} \alpha n_2 n_3 \\ (1-\alpha) n_1 n_3 \\ -n_1 n_2 \end{pmatrix}.$$
