# Supplementary material for: Principal Components Analysis of Population Admixture
Source: PLoS One. 2012 Jul 9;7(7):e40115. doi: 10.1371/journal.pone.0040115 (PMC3392282; doi:10.1371/journal.pone.0040115)
Supplement: Text S3 — Individual-level inference of admixture proportions. (PDF) [file pone.0040115.s009.pdf]

Principal components analysis of population admixture

Jianzhong Ma*, Christopher I. Amos
Department of Genetics, The University of Texas MD Anderson Cancer Center, Houston, Texas, USA
* **E-mail: jzma@mdanderson.org**

Text S3: Individual-level inference of admixture proportions

We first derive the asymptotic form of the reduced eigenequation when all but one of the samples, say N_K, are large. Our starting point is the reduced eigenequation for K populations

$$\begin{pmatrix} -\sum_{l\neq 1} N_l\sigma'_{1l} & N_2\sigma'_{12} & \cdots & N_K\sigma'_{1K} \\ N_1\sigma'_{12} & -\sum_{l\neq 2} N_l\sigma'_{2l} & \cdots & N_K\sigma'_{2K} \\ \vdots & \vdots & \cdots & \vdots \\ N_1\sigma'_{1K} & N_2\sigma'_{2K} & \cdots & -\sum_{l\neq K} N_l\sigma'_{lK} \end{pmatrix} \begin{pmatrix} x_1 \\ x_2 \\ \vdots \\ x_K \end{pmatrix} = \lambda^* \begin{pmatrix} x_1 \\ x_2 \\ \vdots \\ x_K \end{pmatrix},$$

where

$$\begin{aligned} \sigma'_{lk} &= \sigma_{lk} - \sigma_{ak} - \sigma_{al} + \sigma^2_{\bar{c}} \\ \sigma_{ak} &= \frac{1}{N}\left[\sigma^2_k + (N_k-1)\sigma_{kk} + \sum_{m(\neq k)} N_m\sigma_{mk}\right] \\ \sigma^2_{\bar{c}} &= \frac{1}{N^2}\left[\sum_m N_m\sigma^2_m + \sum_m N_m(N_m-1)\sigma_{mm} + 2\sum_{m>n} N_mN_n\sigma_{mn}\right]. \end{aligned}$$

Because $N_k \gg 1$ for $k \neq K$ and $N_K \sim O(1)$,

$$\begin{aligned} N^2\sigma^2_{\bar{c}} &= \sum_{i,j=1}^{K-1} N_iN_j\sigma_{ij} + O(N) \\ N\sigma_{ia} &= \sum_{m=1}^{K-1} N_m\sigma_{mi}. \end{aligned}$$

Therefore,

$$\sigma'_{lk} = \sigma_{lk} - \frac{1}{N}\sum_{m=1}^{K-1} N_m(\sigma_{ml} + \sigma_{mk}) + \frac{1}{N^2}\sum_{m,n=1} N_mN_n\sigma_{mn} + O(1/N).$$

So, the reduced eigenequation has the asymptotic form:

$$\begin{pmatrix} -\sum_{l\neq 1} N_l\hat{\sigma}_{l1} & N_2\hat{\sigma}_{12} & \cdots & 0 \\ N_1\hat{\sigma}_{12} & -\sum_{l\neq 2} N_2\hat{\sigma}_{l2} & \cdots & 0 \\ \vdots & \vdots & \cdots & \vdots \\ N_1\hat{\sigma}_{1K} & N_2\hat{\sigma}_{2K} & \cdots & -\sum_{l\neq K} N_K\hat{\sigma}_{lK} \end{pmatrix} \begin{pmatrix} x_1 \\ x_2 \\ \vdots \\ x_K \end{pmatrix} = \lambda^* \begin{pmatrix} x_1 \\ x_2 \\ \vdots \\ x_K \end{pmatrix}$$

with

$$\hat{\sigma}_{kk'} = \sigma_{kk'} + \frac{1}{N}\sum_{m,l=1}^{K-1} N_mN_l\sigma_{ml} - \frac{1}{N^2}\sum_{m=1}^{K-1} N_m(\sigma_{km} + \sigma_{k'm})$$

for $k, k' = 1, 2, \cdots, K$ and $k \neq k'$. The solutions of this eigenequation can be obtained from those of the asymptotic eigenequation for the first $K-1$ populations:

$$\begin{pmatrix} -\sum_{l\neq 1} N_l\hat{\sigma}_{l1} & N_2\hat{\sigma}_{12} & \cdots & N_{K-1}\hat{\sigma}_{1K-1} \\ N_1\hat{\sigma}_{12} & -\sum_{l\neq 2} N_2\hat{\sigma}_{l2} & \cdots & N_{K-1}\hat{\sigma}_{2K-1} \\ \vdots & \vdots & \cdots & \vdots \\ N_1\hat{\sigma}_{1K-1} & N_2\hat{\sigma}_{2K-1} & \cdots & -\sum_{l\neq K-1} N_{K-1}\hat{\sigma}_{lK-1} \end{pmatrix} \begin{pmatrix} x_1 \\ x_2 \\ \vdots \\ x_{K-1} \end{pmatrix} = \lambda^* \begin{pmatrix} x_1 \\ x_2 \\ \vdots \\ x_{K-1} \end{pmatrix}.$$

There are $K-2$ non-zero eigenvalues, each of which corresponds to an eigenvector given by

$$\begin{pmatrix} x_1 \\ x_2 \\ \vdots \\ x_K \end{pmatrix} = \begin{pmatrix} \vec{X}_{K-1} \\ x_K \end{pmatrix},$$

where $\vec{X}_{K-1}$ is the eigenvector of the $K-1$ populations corresponding to eigenvalue λ and

$$x_K = \frac{\sum_{i=1}^{K-1} x_i N_i \hat{\sigma}_{iK}}{\lambda + \sum_{i=1}^{K-1} N_i \hat{\sigma}_{iK}} = \frac{\sum_{i=1}^{K-1} x_i N_i \hat{\sigma}_{iK}}{\lambda}.$$

The fact that

$$\sum_{i=1}^{K-1} N_i \hat{\sigma}_{iK} = 0$$

can be proved from

$$\hat{\sigma}_{iK} = \sigma_{iK} + \frac{1}{N} \sum_{m,l=1}^{K-1} N_m N_l \sigma_{ml} - \frac{1}{N^2} \sum_{m=1}^{K-1} n_m (\sigma_{im} + \sigma_{Km}).$$

The last large eigenvalue is reduced to zero because of the very small sample size of population K:

$$\lambda_K = \sum_{i=1}^{K-1} N_i \hat{\sigma}_{iK} = 0.$$

This eigenvalue should be of order of the small eigenvalues corresponding to the within-population variation. It is reduced to zero in the asymptotic form.

Now let us suppose that the last population is an admixture of the rest of the populations. Consider the simplest case when $K=3$. When N_1 and N_2 are large and N_3 is very small (like $N_3 = 1$), the non-zero eigenvalue is [1]

$$\begin{aligned} \lambda &= \left[N_1\sigma_1^2 + N_2\sigma_2^2 + N_2(N_1-1)\sigma_{11} + N_1(N_2-1)\sigma_{22} - 2\sigma_{12}N_1N_2\right]/(N_1+N_2) \\ &\approx N_1N_2(\sigma_{11}+\sigma_{22}-2\sigma_{12})/(N_1+N_2) \\ &= 4N_1N_2\left(\Sigma_1^2+\Sigma_2^2-2\Sigma_{12}\right)/(N_1+N_2) \end{aligned}$$

and for the corresponding eigenvector

$$\begin{aligned} x_1 &= -N_2 \\ x_2 &= N_1. \end{aligned}$$

Therefore,

$$\begin{aligned} x_3 &= \left(x_1N_1\sigma'_{13} + x_2N_2\sigma'_{23}\right)/\lambda \\ &= N_1N_2\left(-\sigma'_{13}+\sigma'_{23}\right)/\lambda. \end{aligned}$$

It is straightforward to show that

$$-\sigma'_{13} + \sigma'_{23} = 4(N_1\alpha_2 - N_2\alpha_1)\left(\Sigma_1^2+\Sigma_2^2-2\Sigma_{12}\right).$$

We thus have

$$x_3 = N_1\alpha_2 - N_2\alpha_1 = x_1\alpha_1 + x_2\alpha_2,$$

where $\alpha_1{:}\alpha_2 = \alpha{:}(1-\alpha)$ is the admixture proportion. This means that the eigenvector pattern still follows the same rule according to the admixture proportion even if the admixed population is small as long as the parental populations have a large sample size.
